# *In Vivo* Hypocholesterolemic Effect of MARDI Fermented Red Yeast Rice Water Extract in High Cholesterol Diet Fed Mice

**DOI:** 10.1155/2014/707829

**Published:** 2014-06-16

**Authors:** Swee Keong Yeap, Boon Kee Beh, Joan Kong, Wan Yong Ho, Hamidah Mohd Yusof, Nurul Elyani Mohamad, Aminuddin bin Hussin, Indu Bala Jaganath, Noorjahan Banu Alitheen, Anisah Jamaluddin, Kamariah Long

**Affiliations:** ^1^Institute of Bioscience, Universiti Putra Malaysia, 43400 Serdang, Selangor, Malaysia; ^2^Department of Bioprocess Technology, Faculty of Biotechnology and Biomolecular Sciences, Universiti Putra Malaysia, 43400 Serdang, Selangor, Malaysia; ^3^Aning Biotech Corporation Sdn Bhd., 13G & 13-1, Jalan LJ3, Lembah Jaya Industrial Park, 68000 Ampang, Selangor, Malaysia; ^4^School of Biomedical Sciences, The University of Nottingham, Malaysia Campus, Jalan Broga, 43500 Semenyih, Selangor, Malaysia; ^5^Department of Cell and Molecular Biology, Faculty of Biotechnology and Biomolecular Sciences, Universiti Putra Malaysia, 43400 Serdang, Selangor, Malaysia; ^6^Department of Bioprocess Biotechnology, Malaysian Agriculture Research Development Institute, 43400 Serdang, Selangor, Malaysia; ^7^Biotechnology Research Centre, Malaysian Agriculture Research Development Institute, 43400 Serdang, Selangor, Malaysia

## Abstract

Fermented red yeast rice has been traditionally consumed as medication in Asian cuisine. This study aimed to determine the *in vivo* hypocholesterolemic and antioxidant effects of fermented red yeast rice water extract produced using Malaysian Agricultural Research and Development Institute (MARDI) *Monascus purpureus* strains in mice fed with high cholesterol diet. Absence of monacolin-k, lower level of *γ*-aminobutyric acid (GABA), higher content of total amino acids, and antioxidant activities were detected in MARDI fermented red yeast rice water extract (MFRYR). *In vivo* MFRYR treatment on hypercholesterolemic mice recorded similar lipid lowering effect as commercial red yeast rice extract (CRYR) as it helps to reduce the elevated serum liver enzyme and increased the antioxidant levels in liver. This effect was also associated with the upregulation of apolipoproteins-E and inhibition of Von Willebrand factor expression. In summary, MFRYR enriched in antioxidant and amino acid without monacolin-k showed similar hypocholesterolemic effect as CRYR that was rich in monacolin-k and GABA.

## 1. Introduction

Red yeast rice, a product of* Monascus* fermentation, has traditionally been used as a medicine (to improve digestion and blood circulation), preservative (Chinese bean curd/cheese, meat, fish, etc.), and coloring agent and inoculant for alcoholic beverages (*Anchu*,* somsu*) in Asian countries since ancient time [1]. It can be produced through either the submerged or the solid state fermentation system. Rice is the common fermentation medium [2] to produce* Monascus* fermentation products [3] besides other agricultural products including dioscorea, cassava, sweet potato, potato [4], barley [5], mixture of rice and garlic [6], soybean [7], corn meal, peanut meal, coconut residue, and soybean meal. Monacolin-k (that is identical to lovastatin) was the most commonly standardized monacolin compound in fermented red yeast rice for its lipid lowering property via inhibition of the 3-hydroxy-3-methylglutaryl-coenzyme A reductase [8]. Other secondary metabolites including *γ*-aminobutyric acid (GABA), dimerumic acid, and monascin, which are potential hypotensive, antioxidant, and anti-inflammation agents, were also reported as being present in fermented red yeast rice [4, 9, 10].

Over the past decades, red yeast rice has gained drastic attention and sales due to its use as statin alternative therapy for hyperlipidemia and dyslipidemia management [11]. Clinical studies have reported that red yeast rice can significantly reduce lipid level and prevent recurrence of coronary events in human subjects [12, 13]. However, the Food and Drug Administration (FDA, USA) issued a consumer warning to avoid online promotion of commercial red yeast rice products in August 2007 due to the presence of monacolin-k, which is biosimilar to the active pharmaceutical ingredient lovastatin [14]. Use of statin drug has been reported to cause some side effects such as headache, dizziness, rash, stomach discomfort, hepatic dysfunction, muscle weakness, or even rhabdomyolysis symptoms [15]. Although many studies have proven the potential lipid lowering properties of* Monascus* fermented red yeast rice, the effects of fermented red yeast rice water extract produced using the MARDI* Monascus purpureus* strains, which did not contain monacolin-k and their derivatives, are not yet to be determined and compared with fermented red yeast rice extract that contains monacolin-k. Hence, the aim of this study was to determine and compare the hypocholesterolemic and antioxidant effects of fermented red yeast rice water extract (MFRYR) produced from the MARDI* Monascus purpureus* strains with commercial red yeast rice extract (CRYR) in high cholesterol diet mice model* in vivo*.

## 2. Materials and Methods

### 2.1. Materials

Cholesterol, 94%, was purchased from Sigma Aldrich (USA). Commercial red yeast rice extract (CRYR) was purchased from the local pharmacy store. Hypoxanthine, xanthine oxidase, superoxide dismutase, Folin-Ciocalteu reagent, aluminium chloride, sodium nitrate, ascorbic acid, and gallic acid were obtained from Sigma Aldrich (USA). Griess reagent was from Invitrogen (USA). All solvents used were of analytical reagent or HPLC grade.* Monascus purpureus* strain inoculumwas obtained from MARDI's culture collection center.

### 2.2. Preparation of MARDI Fermented Red Yeast Rice Water Extract (MFRYR) Using* Monascus purpureus* Strains

Broken rice (1000 g) was washed 6 times and soaked in cold water at room temperature for 18 h. The soaked broken rice was washed, autoclaved at 121°C for 20 min, and cooled down to room temperature. The broken rice was then inoculated with MARDI* Monascus purpureus* strains and incubated aerobically at 32°C for 16 days. It was then harvested, oven-dried, ground into powder, and extracted with deionized water. The MARDI* Monascus purpureus* strains fermented red yeast rice water extract (MFRYR) was lyophilised using a Virtis BenchTop freeze dryer (SP Industries, Inc., USA) and stored in a 4°C chiller until analysis. Nonfermented rice, MFRYR, and commercial red yeast rice extract (CRYR) were subjected to monacolin-k and GABA quantification using the UPLC method. Monacolin-k analysis was conducted using the Acquity UPLC system (Waters Corp., USA) equipped with a RP-18 column (1.7 *μ*m, 2.1 × 100 mm, Waters Acquity, USA) and gradient mobile phase of 100% acetonitrile (Eluent A) and 0.1% trifluoroacetic acid (Eluent B). The linear gradient mode was performed (flow rate at 0.2 mL/min) from 35% to 75% Eluent A for 7.8 min and maintained at 75% Eluent A for another 3.2 min before being reverted back to 35% Eluent A for 0.8 min. Total analysis time was set for 14 min with an injection volume of 1 *μ*L and column temperature of 30°C. Monacolin-k was detected using a Photodiode array (PDA) detector at 237 nm with the detection range between 210 and 350 nm. On the other hand, the concentration of GABA and amino acids was determined according to Ali et al. [16]. The* in vitro* antioxidant effects of nonfermented rice, MFRYR, and CRYR were compared using total phenolic content and Ferric reducing power (FRAP) tests [17].

### 2.3. Hypocholesterolemic Study

Male Balb/c mice (8 weeks old, average body weight of 25 ± 2 g), obtained from the Animal Housing Department, Institute of Bioscience, Universiti Putra Malaysia, were used for the experiments below. The mice were kept in prebeded plastic cages in controlled conditions of 22 ± 3°C and standard 12 hours of day/dark light cycles with food and water* ad libitum*. The procedures for this study were carried out according to the guidelines of the National Institute of Health for the Care and Use of Laboratory Animals (ref.: UPM/FPV/PS/3.2.1.551/AUP-R2). Male Balb/c mice were randomly assigned into five groups with eight animals each, namely:

 group 1: normal control, mice (p.o.) receiving normal saline daily only;
 group 2: negative control, mice (p.o.) receiving cholesterol at 1000 mg/kg concentration and normal saline daily only;
 group 3: MFRYR treated group, receiving cholesterol at 1000 mg/kg concentration and 6 mg/kg of body weight MFRYR daily;
 group 4: MFRYR treated group, receiving cholesterol at 1000 mg/kg concentration and 60 mg/kg of body weight MFRYR daily;
 group 5: CRYR treated group, receiving cholesterol at 1000 mg/kg concentration and 60 mg/kg of body weight CRYR daily.

All groups (except group 1) were preincubated with cholesterol 1000 mg/kg body weight (p.o.) for 8 weeks and continued with cholesterol and the respective extract treatments for another 2 weeks. On the last day of the experiment, the mice were sacrificed. Blood and liver were collected for the following analyses.

#### 2.3.1. Biochemical Assays of Lipid and Liver Profiles in Serum

The lipid profile (total cholesterol, TAG, LDL, and HDL) and liver profile (ALT, ALP, and AST) in serum were measured using a biochemical analyzer (Hitachi 902 Automatic Analyzer) and adapted reagents from Roche (Germany) [16].

#### 2.3.2. Liver Histopathological Evaluation

Liver was removed, fixed, and stained in haematoxylin and eosin (H&E) [16]. The histopathological alterations of the liver from different groups were observed using a bright-field microscope (Nikon, Japan).

#### 2.3.3. *In Vivo* Antioxidant Assays for Mice Liver Homogenate

Liver homogenates were prepared by meshing the harvested liver in ice-cold PBS followed by homogenization and centrifugation at 2000 rpm and 4°C for 5 minutes. The supernatants collected were subjected to superoxide dismutase (SOD) and malondialdehyde (MDA) assays [16]. One unit of SOD was calculated as the amount of protein needed to achieve 50% inhibition and was expressed as unit SOD/mg protein while MDA activity was expressed as nmol MDA/g protein.

#### 2.3.4. PCR Array on Atherosclerosis Related Gene Expression in Blood

Blood from group 2, group 4, and group 5 was collected and subjected to RNA extraction using the RNeasy mini kit (Qiagen, USA). cDNA synthesis and mouse atherosclerosis RT^2^ Profiler PCR array (SABiosciences, USA) were performed using an iCycler iQ real-time PCR system (Bio-Rad, USA) according to the manufacturer's protocols. The results were normalized with the five housekeeping genes which were included in the kit and the relative fold change was determined by dividing the normalized data of the genes from samples of groups 4 and 5 with the normalized data of the genes from the untreated group 2.

### 2.4. Statistical Analysis

All quantitative measurements were conveyed as mean ± S.D. Analyses were performed using one-way analysis of variance (ANOVA) and the group means were compared by Duncan test. *P* values <0.05 were considered as statistically significant.

## 3. Results and Discussion

### 3.1. *In Vitro* Monacolin-k, GABA, Amino Acid, Total Phenolic Contents, and Antioxidant Effects (FRAP Activity) of MFRYR

Fermentation using* Monascus *spp. was recorded with enhanced levels of nutritious compounds including *γ*-aminobutyric acid (GABA) and monacolin-k. However, monacolin-k was found to be associated with several side effects including hepatotoxicity [15] and it was advised by the FDA for it is not to be present in commercial red yeast rice supplements [14]. A previous study has reported that alterations of fermentation conditions including fermentation temperature, aeration, and nutritional factors and method (submerged and solid state fermentations) were able to prevent the production of monacolin-k by* M. purpureus* without altering the hypocholesterolemic effect of this red yeast rice [18]. For example, Ajdari et al. [18] suggested that the hypocholesterolemic effect of this fermented product without monacolin-k was contributed by other bioactive compounds. In this study, we have evaluated the monacolin-k, GABA, total amino acid, and antioxidant levels in the water extract of MARDI's* Monascus purpureus* fermented red yeast rice (MFRYR). Chromatographic analyses showed that monacolin-k, GABA, and amino acid (both free and essential) were not detected in the nonfermented rice sample. The commercial fermented red yeast rice (CRYR) sample was detected to have high level of monacolin-k (182.61 ± 0.02 *μ*g/g) but it was not found in the MFRYR sample. GABA content was recorded to be 3.4- fold higher in the CRYR sample as compared to MFRYR sample (Table 1). Wang et al. [19] had demonstrated that the alteration of the fermentation process could modulate the production of monacolin-k and GABA in a similar trend by using* M. purpureus*. Thus, it was not surprising to find that MFRYR which did not contain monacolin-k had a lower GABA concentration since these two compounds were always produced with similar trends during fermentation [19]. On the other hand, MFRYR recorded the highest total free and essential amino acids as compared to CRYR. Nevertheless, both nonfermented rice and MFRYR were recorded to have 2-fold higher phenolic content and antioxidant activities in the FRAP antioxidant test when compared to CRYR. These indicated that MARDI's* Monascus purpureus* strains were able to increase the antioxidant level during the fermentation process. The results showed that the MFRYR contained higher levels of total amino acid (free and essential) and antioxidants as compared to commercial red yeast rice extract (CRYR) and nonfermented rice (Table 1).

### 3.2. *In Vivo* Hypocholesterolemic and Antioxidant Effects of MFRYR

**Figure 1:**
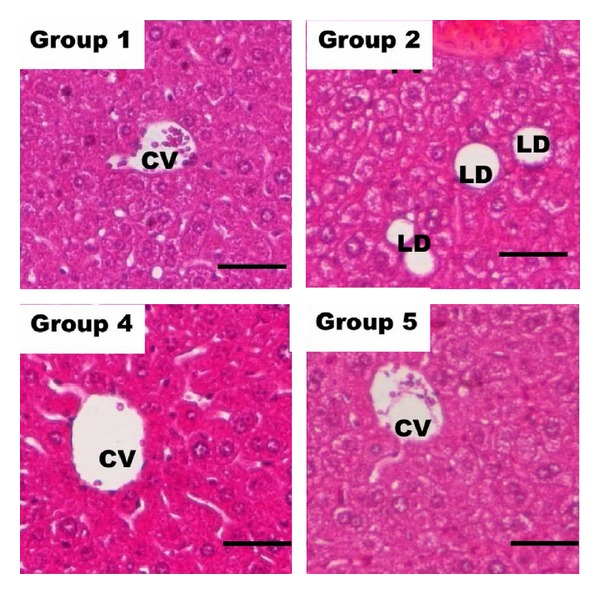
Representative histological micrograph of liver sections from groups 1, 2, 4, and 5 of 14-day treatment stained with H&E (×100, Bar = 50 *μ*m). Only untreated group (group 2) showed lipid droplet in the histological section. CV = central vein, LD = lipid droplet, and PV = portal vein.

In this experiment, high cholesterol mice were induced by feeding cholesterol p.o. at concentration of 1000 mg/kg body weight daily for continuously 8 weeks before proceeding with cholesterol and extract treatments for another 2 weeks. The results showed significant reductions of total cholesterol, triglycerides (TAG), and low density lipoprotein (LDL) levels and significant increment of high density lipoprotein (HLD) among extract treatment groups as compared to the untreated control group 2 (Table 2). MFRYR had a dose-dependent hypocholesterolemic effect whereby hypercholesterolemic mice treated with higher concentration (60 mg/kg body weight) showed more significant reduction of serum lipid profiles (Table 2). Besides, MFRYR had comparable hypocholesterolemic properties comparing to CRYR where treatment with 60 mg/kg body weight of MFRYR and CRYR showed approximate 22% of total cholesterol, 30% of TAG, and 55% of LDL reduction. In terms of HDL level, the highest increment (~36%) was recorded in CRYR treatment group. Besides the serum lipid profile, liver histopathology observation was carried out in this experiment.  Figure 1 showed the histology of the liver sections for groups 1, 2, 4, and 5. Lipid droplets were only observed in livers of untreated mice, which indicated that all treatments were able to reduce fat accumulation in the livers of the high cholesterol diet mice. Besides monacolin-k, GABA which was reported as one of the major active ingredients in germinated brown rice could also contribute to the hypocholesterolemic effect [20]. In this study, although monacolin-k was not detected and GABA was 3.4-fold lower, MFRYR recorded equally good hypocholesterolemic effect as CRYR in a dosage dependent manner (Table 2 and Figure 1). This effect may be contributed by the increased levels of phenolic content and total amino acids in MFRYR. Afonso et al. [21] and Børsheim et al. [22] reported the potential use of phenolic compounds and amino acid supplementation to reduce serum lipid profile which in turn complemented our observations in this study. More importantly, phenolic compounds have been reported as one of the most important dietary bioactive compounds that contributed to the antioxidant effect [21].

In general, consumption of a high fat diet could contribute to the formation of fatty liver and subsequently elevated the liver enzyme levels as observed in the untreated high cholesterol diet group (Table 2). All extract treatments showed different degrees of liver enzyme recoveries when compared to the untreated cholesterol control (group 2). MFRYR at both concentrations managed to recover the elevated liver enzymes to lower levels in the dosage dependent manner. Nonetheless, the elevated liver enzyme levels in the MFRYR treatment groups at both concentrations were still higher than the normal control (group 1). On the other hand, the CRYR group was found to have higher serum liver enzyme profile, which was close to the untreated cholesterol control (group 2). Recovery from liver damage was always correlated to the antioxidant, lipid peroxidation, and nitric oxide level in the liver [16]. Similar to the serum liver profile, liver homogenates from the untreated cholesterol control and CRYR treated group were recorded to have the higher lipid peroxidation and the lower SOD antioxidant enzyme level. On the other hand, MFRYR was able to reduce lipid peroxidation and increase SOD level in a dosage dependent manner (Table 3). Red yeast rice produced by* M. purpureus* via fermentation was also found to be associated with higher* in vitro* and* in vivo* antioxidant effects [23]. The degree of enhanced antioxidant level may differ based on the initial antioxidant level found in the raw material used [24]. In this study, nonfermented rice was found to contain higher antioxidant level (total phenolic content and FRAP antioxidant activity) than CRYR. Thus, it was not surprising to observe that MFRYR with further enriched antioxidants than the nonfermented rice had recorded much higher* in vitro* (Table 1) and* in vivo* (Table 3) antioxidant activities. Unlike MFRYR, higher level of serum liver enzyme profile and lower level of liver antioxidant in CRYR treated group might be due to the presence of the potentially hepatotoxic monacolin-k [15] and the lower level of antioxidant (Table 1) compared to MFRYR.

High level of serum cholesterol is often associated with increased risk of atherosclerosis [25]. To understand the possible mechanism of the hypocholesterolemic effect by MFRYR and CRYR, RNA was extracted from blood and subjected to PCR array for evaluation of atherosclerosis related gene expressions. Only fold changes greater than ±2 were recorded in Table 4 as significant regulated genes in comparison to the untreated cholesterol group (group 2). MFRYR only showed significant upregulation of* Apoe* and downregulation of* Vwf* gene expressions. On the other hand, CRYR was able to significantly upregulate* Abca1* and* Apoe* (~3-fold) while downregulating* Ccl2*,* Cd44*,* Fn1*,* Itga2*,* Lpl*,* Msr1*,* Npy* (~4-fold),* Ptgs1*,* Selp*,* Tgfb2*,* Tnf*,* Vcam1*, and* Vwf* (~4-fold). The regulations of* Apoe* and* Vwf* by MFRYR showed similar trends as CRYR but at lower magnitudes. Furthermore, MFRYR also showed regulations of* Abca1*,* Ccl2*,* Cd44*,* Fn1*,* Itga2*,* Lpk*,* Msr1*,* Npy*,* Ptgs1*,* Selp*,* Tgfb2*,* Tnf*, and* Vcam1* with similar trends as CRYR but with fold changes of less than 2 (results not shown). The deficiency of* ApoE *is related to the elevation of blood cholesterol level and subsequently leads to increased risk of atherosclerosis [26]. On the other hand,* Vwf* is a procoagulant glycoprotein which has been widely used as an indicator of endothelial damage during atherosclerosis [27]. These two genes have been significantly regulated by both MFRYR and CRYR. More atherosclerosis related genes were regulated in the CRYR treated mice indicating that MFRYR and CRYR which contained different types of active metabolites possessed hypocholesterolemic effects via different mechanisms. As indicated by Table 1, total phenolic contents of MFRYR were 20 times higher than CRYR. Previous study has reported that common polyphenol that is in grape seed, which were gallic acid and catechin, inhibited the pancreatic cholesterol esterase and reduced solubility of cholesterol in micelles thus slowing the adsorption of cholesterol [28]. Thus, polyphenols that are present in MFRYR may also utilize similar mechanism to reduce adsorption of cholesterol in this study. However, further detailed transcriptome analysis on the hypercholesterolemic mice treated with amino acid and phenolic compounds isolated from MFRYR is needed to have a better understanding of the different hypocholesterolemic regulations between MFRYR which does not and CRYR which does contain monacolin-k.

## 4. Conclusion

MFRYR contained higher free amino acid (111-fold) and total phenolic level (20 times) comparing to unfermented rice and CRYR. Furthermore, MFRYR reduced the cholesterol level similarly and more effectively enhanced the antioxidant level in hypercholesterol mice comparing to CRYR. Atherosclerosis related gene expression study proposed that MFRYR may differentially regulate hypocholesterolemic effect comparing to CRYR. These results concluded that MFRYR that is free from monacolin-k possesses good and comparable hypocholesterolemic properties as CRYR with better hepatoprotective effect contributed by the enhanced antioxidants present in MFRYR.

## Figures and Tables

**Table 1:** Monacolin-k, GABA, total amino acid (free and essential), total phenolic content, and FRAP antioxidant activity of nonfermented rice, MARDI fermented red yeast rice water extract (MFRYR), and commercial red yeast rice extract (CRYR).

	Nonfermented rice	MFRYR	CRYR
Monacolin-k (µg/g sample)	ND	ND	182.61 ± 0.02
GABA (g/100 g sample)	ND	0.14 ± 0.01	0.48 ± 0.03
Total free amino acid (g/100 g sample)	ND	3.33 ± 0.54	0.03 ± 0.01
Total essential amino acid (g/100 g sample)	ND	1.32 ± 0.26	0.03 ± 0.01
Total phenolic content (µg GAE/mg sample)	7.60 ± 0.10	16.20 ± 0.10	0.81 ± 0.10
FRAP (mg ascorbic acid equivalent (AAE)/g sample)	29.80 ± 0.01	74.04 ± 0.02	5.40 ± 0.02

ND: not detected; values were mean ± standard deviation of three independent experiments.

**Table 2:** Blood serum lipid and liver profile.

Treatment	Cholesterol (mg/dL)	Triglyceride (mg/dL)	LDL (mg/dL)	HDL (mg/dL)	ALT (U/L)	ALP (U/L)	AST (U/L)
Group 1 (*n* = 8)	115.05 ± 10.53∗	177.11 ± 13.19∗	39.00 ± 2.39∗	47.19 ± 3.17∗	62.50 ± 3.55∗	90.13 ± 4.45∗	120.42 ± 13.05∗
Group 2 (*n* = 8)	230.47 ± 15.99	264.33 ± 23.40	113.85 ± 3.90	56.55 ± 4.29	277.20 ± 9.76	127.83 ± 4.29	320.13 ± 30.71
Group 3 (*n* = 8)	201.72 ± 24.57	202.03 ± 25.86	62.40 ± 1.56∗	64.16 ± 1.95∗	155.33 ± 9.73∗	106.75 ± 4.69∗	170.49 ± 31.69∗
Group 4 (*n* = 8)	179.85 ± 10.12∗	171.30 ± 12.60∗	44.28 ± 4.33∗	70.59 ± 6.17∗	173.13 ± 5.58∗	105.80 ± 1.08∗	181.16 ± 17.59∗
Group 5 (*n* = 8)	181.07 ± 11.06∗	176.22 ± 24.08∗	52.26 ± 3.12∗	77.42 ± 4.29∗	263.58 ± 5.08	137.00 ± 3.70	424.42 ± 43.07∗

Values were mean ± standard deviation of 8 animals in each group and significant difference indicated by ∗(*P* < 0.05) was determined using ANOVA followed by Duncan's multiple range test.

**Table 3:** Liver homogenate lipid peroxidation and SOD enzyme levels.

Treatment	MDA (nM MDA/mg sample)	SOD (unit SOD/mg sample)
Group 1 (*n* = 8)	0.72 ± 0.15∗	0.90 ± 0.12∗
Group 2 (*n* = 8)	2.21 ± 0.13	0.60 ± 0.01
Group 3 (*n* = 8)	1.11 ± 0.20∗	0.72 ± 0.09∗
Group 4 (*n* = 8)	0.90 ± 0.03∗	0.83 ± 0.03∗
Group 5 (*n* = 8)	1.46 ± 0.21∗	0.70 ± 0.19∗

Values were mean ± standard deviation of 8 animals in each group and significant difference indicated by ∗(*P* < 0.05) was determined using ANOVA followed by Duncan's multiple range test.

**Table 4:** Relative expression of atherosclerosis related gene in blood of 60 mg/kg body weight of MFRYR (group 4) and CRYR (group 5) treated mice as compared to the untreated mice (group 2).

Genes	Group 4 (MFRYR 60 mg/kg body weight)	Group 5 (CRYR 60 mg/kg body weight)
	Relative expression (fold change)	
ATP-binding cassette transporter 1 (Abca1)	N.S	2.00 ± 0.02
Apolipoproteins-E (ApoE)	**2.66 ± 0.11**	**3.68 ± 0.09**
Chemokine (C-C motif) ligand 2 (CCL2)	N.S	−2.00 ± 0.03
CD44	N.S	−2.01 ± 0.01
Fibronectin (Fn1)	N.S	−2.03 ± 0.01
Integrin, alpha 2 (ITGA2)	N.S	−2.00 ± 0.03
Lipoprotein lipase (LPL)	N.S	−2.00 ± 0.02
Macrophage scavenger receptor 1 (MSR1)	N.S	−2.01 ± 0.04
Neuropeptide Y (Npy)	N.S	−3.85 ± 0.01
Prostaglandin-endoperoxide synthase 1 (PTGS1)	N.S	−2.00 ± 0.03
Selectin P (SELP)	N.S	−2.00 ± 0.05
Transforming growth factor, beta 2 (TGFB2)	N.S	−2.01 ± 0.02
Tumor necrosis factor (TNF)	N.S	−2.00 ± 0.01
Vascular cell adhesion molecule 1 (Vcam1)	N.S	−2.00 ± 0.01
Von Willebrand factor (Vwf)	**−3.00 ± 0.13**	**−4.24 ± 0.12**

N.S.: not significant, only fold change greater than ±2 was recorded and presented as significant results.

## Abbreviations

CRYR: Commercial red yeast rice extract
MFRYR: MARDI* Monascus purpureus* strain fermented red yeast rice water extract
GABA: *γ*-aminobutyric acid
TAG: Triglycerides
LDL: Low density lipoprotein
HDL: High density lipoprotein
ALT: Alanine transaminase
ALP: Alkaline phosphatase
AST: Aspartate aminotransferase
ApoE: Apolipoproteins-E
Vwf: Von Willebrand factor.
